# Research on Innovation Ecosystem of Dairy Industry Cluster Based on Machine Learning and Improved Neural Network

**DOI:** 10.1155/2022/4509575

**Published:** 2022-04-04

**Authors:** Yang Hui, Yanni Jiao, Can Cui, Ke Ma

**Affiliations:** College of Economics and Management, Northeast Agricultural University, Heilongjiang, Harbin, China

## Abstract

With the rapid development of global economy, the industrial cluster has become the new trend of world economic development. So far, the industrial cluster mode with space as the main division has been formed. The cooperation of industrial clusters is dynamic. The industries in the cluster cooperate with each other for mutual benefit and win-win, occupying a place in the fierce market competition. In addition, the industrial cluster is also conducive to strengthen the international economic division of labor and points out the direction of regional industrial transfer. China is now in a critical period of economic development; the industrial cluster plays an important role in China's industrial transformation and economic development. At present, the most common regional development mode in China is industrial cluster. The emergence of industrial cluster accelerates the development of industrial regional economy and the balance of industrial layout. Industrial clusters occupy a key position in China's economic industry chain, and the development and change in industrial clusters will directly affect the development of the entire industrial chain. This study first simulates the evolution path of industrial cluster and then establishes the relevant data model. Finally, through repast simulation, it puts forward conclusions and suggestions according to the verification results. The construction of dynamic model can realize the simulation of industrial cluster theory. According to the simulation results, we can find that the ecosystem in different stages will produce different characteristics; the formation and evolution of industrial clusters are actually the epitome of market development. In this process, the government guidance and market regulation are needed to accelerate the formation of industrial cluster ecosystem and increase the scale of industrial cluster.

## 1. Introduction

After the reform and opening up, China's industrial clusters have developed rapidly. On the one hand, the economic development after the reform and opening up relies more on the development of regional economy, and the development of industries in the same region is basically the same, which is conducive to the formation of industrial clusters; on the other hand, there is a highly specialized division of labor and close industrial ties between different industries. It has laid a rich foundation for the formation of industrial clusters [1]. At present, China has formed a relatively mature industrial cluster ecosystem. Different industries cooperate with each other and occupy a dominant position in the market. In the development process of China's industrial clusters, each stage has different changes. Industrial clusters should also make corresponding changes according to the characteristics of different stages, follow the trend of market development, and maintain the advantages of industrial clusters. So far, the common idea in academic circles is that China's industrial clusters will develop in the direction of cluster ecosystem, thus forming the advantage of market competition [2]. However, the current academic research on the cluster ecosystem is not deep enough, and the social understanding of the cluster ecosystem only stays on the concept of “ecological rent,” but there is no accurate understanding of the specific concepts of cluster ecosystem and “ecological rent.” This is the focus of our future research; we need to popularize the concept of cluster ecosystem to the whole society.

The theory of multi-industry cluster and social organization is a kind of complex system. Among the organizations and institutions that make up the cluster, microenterprises occupy the most important position. The formation of industrial clusters does not depend on the drive of a single enterprise, but requires the collaborative development of enterprises in the industry [3]. To form a mature and orderly industrial cluster structure, we should constantly innovate and update the cluster system and related decisions to ensure that the industrial cluster can keep up with the pace of the times. It is also very important for industrial clusters to make correct decisions. Good decisions can promote the rapid development of industrial clusters, even change the macro-state of industrial clusters, and promote the sustainable development of industries. Therefore, before carrying out the policy, we should fully consider whether the policy meets the actual requirements and whether the policy is rigorous enough. Only after the rationality of the policy is fully determined, the policy can be issued. In addition, through the establishment of industrial cluster simulation model to test whether the various factors affecting the development of industrial cluster are reasonable.

## 2. Related Work

The idea of industrial cluster was first put forward by Adam Smith. Literature [4] thinks that division of labor is very important in industrial cluster. Only by determining the role of each part of industrial cluster, we can give full play to the advantages of industrial cluster and form scale economy. The development of industrial clusters should not only pay attention to the cooperation between various departments but also pay attention to the refinement of functions. On the research of industrial clusters, the theory of industrial location proposed by literature [5] and the theory of industrial location proposed by literature [6] have become the important ideas for the development of industrial clusters, and more and more studies have been carried out with these two ideas. However, foreign scholars only focus on the causes of industrial clusters and do not conduct in-depth research on the follow-up development of industrial clusters. Research: in addition, most of the foreign research on industrial cluster is based on the theory of industrial cluster. Literature [7] and others began to study the theory of technology spillover, and literature [8] carried out the research on knowledge management of industrial clusters. In the research, we should first study different cluster organizations separately. Literature [9] classifies the research focus as the knowledge resources of industrial clusters. At present, the research focus of industrial clusters is the research integration between different schools of industrial clusters and the theoretical knowledge in industrial clusters. This change shows that the academic research on industrial clusters has begun to transition from theory to practice, which is a qualitative leap for industrial clusters.

Literature [10] puts forward that in the whole industrial cluster ecosystem, some ecological factors related to the industrial cluster ecosystem jointly promote the development and progress of the cluster industry, promote the formation of the cluster effect within the industry, and promote the cooperation of all parts of the cluster. In reference [11], the industrial cluster ecosystem should not be a simple industrial collection, it is formed in the long-term evolution process, and the regional economic ecosystem is a dynamic system. In the development of industrial clusters, we should pay attention to the establishment of industrial brands to form competitive advantages. Literature [12] believes that the emergence of brand effect is with the development of industrial clusters, and when the industrial clusters develop to a certain extent, it will promote the formation of brands within the industry, thus producing brand effects externally. Literature [13] has proved that for industrial clusters, transformation is the only way, and the key to achieve transformation is to maintain the competitive advantage within the cluster. Literature [14] analyzes the impact of network on industrial clusters from the perspective of network. The analysis results show that the use of network in the cluster can strengthen the relationship among various parts of the cluster and can also improve the competitive advantage of individual enterprises in the cluster. According to the resource-based theory proposed in reference [15], heterogeneous resources of enterprises can be divided into tangible and intangible resources. Although the existence forms of these two resources are different, they are indispensable resources in the process of cluster. These resources are the source of competitiveness and creativity of enterprises and play an irreplaceable role. Literature [16] believes that rich resources can ensure the dominant position of enterprises in the competition. In literature [17] for the enterprises in the cluster, it is very important whether they have their own rooted resources, which is the key element to distinguish enterprises from other enterprises in the cluster, and the core and key in the cluster ecosystem. Literature [18] has proved the importance of heterogeneous resources to enterprises. Heterogeneous resources can catalyze the emergence of competitive advantages of enterprises in clusters. In addition to promoting the development of individual enterprises, they can also promote the common development of industrial clusters and even the entire industrial chain. According to the literature [19], competition and cooperation are the two basic ways of industrial cluster formation. The existence of these two ways ensures the continuous creation of new things within the industrial cluster, with continuous vitality and sustainable development. The industrial cluster ecosystem is similar to the natural ecosystem, but the competition between enterprises cannot be compared with the relationship in the natural ecosystem. The competition between enterprises includes both struggle and cooperation, which is different from the single competition in nature. In fact, competition is the best cooperation. Based on the theory of cooperative competition, literature [20] confirmed that orderly competition in the cluster can promote the common development of enterprises and clusters and make the enterprises in the cluster improve their competitiveness in the market. Literature [21] holds that competition is necessary for cooperation. In fact, cooperation is developed from competition, and cooperation is a consensus reached in the process of competition. According to the niche theory, literature [22] holds that market competition is actually a struggle for innovation ability, especially for enterprises of the same nature, and if they want to stand out among the same kind, they must have strong innovation ability and seek new profit growth points; in addition, competition is necessary for the development of clusters, and competition can promote cooperation and division of labor among enterprises. At the same time, it will promote the exchange and integration of different enterprises in the same industry chain. In reference [23], the industrial cluster ecosystem is a dynamic system. In the long-term development process, each component of the ecosystem realizes common survival and coevolution, aiming at improving the overall competitiveness and making common progress.

Agent theory was first proposed in the field of artificial intelligence. As mentioned in literature [24], so far, agent theory has not only existed in the field of artificial intelligence, but also applied to various fields of society. For different fields, the meaning of agent theory is different. Different fields will give different meanings to agent theory to meet the needs of this field. Therefore, it is very difficult to determine the unified definition of agent theory. Although different fields have different definitions of agent theory, each domain has some common characteristics, such as autonomy, intelligence, autonomy, and sociality.

## 3. Technology and Theory of the Dairy Industry Cluster Ecosystem

### 3.1. Modeling Method of the Multiagent System

Multiagent system comes from the field of artificial intelligence and has been applied to many fields. MAS is a system composed of multiple agents. MAS can achieve the task goal through the cooperation between agent systems. MAS is actually a complex joint system. This system can realize data simulation and solve the actual problems.

Before using multiagent system for research, we should first determine the specific role of each element in the system and clarify the relationship between different elements; secondly, we should extract the key elements of the system, establish the agent model of the system, and set the rule conditions of the agent model; finally, after the establishment of the agent model, the model is run and data statistics and analysis are carried out, and the analysis results with the actual results are compared.

In MAS system, the agent is defined as individual, and the relationship in MSA system can be regarded as the static relationship model between different individuals; in the construction of multiagent class, the interaction and cooperation are carried out through the establishment of each class to form a model for operation and solve each level and task in the system.

### 3.2. Quantitative Model of Competition Cooperation Interaction Game of Industrial Cluster

#### 3.2.1. Basic Assumptions of the Model

(i) Suppose that there are only two enterprises (1, 2), and the two enterprises are substitutes. The total supply and demand function in the market is as follows:
  (1) $$\begin{array}{c} Q=q_{1}+q_{2},\\ p\left(Q\right)=p\left(q_{1}+q_{2}\right). \end{array}$$
(i) P is the price, and Q(p) is the original demand function. The two companies here have the same unit cost *c* and total output Q. Suppose
  (2) $$\begin{array}{c} p\left(Q\right)=A-Q,\\ C_{i}\left(Q\right)=q_{i}c. \end{array}$$
(i) Profit *R* is paid by the enterprise itself, and it is a function of output *Q*.

#### 3.2.2. Game Model Analysis

We can get the model we want by comparing the companies in a single time. According to the model obtained, the profit of the two companies is calculated as follows:

(3) $$\begin{array}{c} R_{1}=R_{2}=Q*P=\frac{A-c}{4}\times \left(P-c\right)\frac{{\left(A-c\right)}^{2}}{8}. \end{array}$$

The profit of a single enterprise is increased, and the scale is expanded. The profit obtained is as follows:

(4) $$\begin{array}{c} R_{1}=q_{1}\left(p-c\right)=q_{1}\left(A-Q-c\right)=q_{1}\left[A-\left(q_{1}+q_{2}\right)-c\right]. \end{array}$$

The optimized first-order condition is as follows:

(5) $$\begin{array}{c} \frac{\partial R_{1}}{\partial R_{2}}=A-\left(2q_{1}+q_{2}\right)-c=0. \end{array}$$

The relationship between the profit and output of the company can be obtained. In the case of other companies also cooperating,

(6) $$\begin{array}{c} q_{2}=\frac{A-c}{4},\\ q_{1}=\frac{3\left(A-c\right)}{8}. \end{array}$$

Thus,

(7) $$\begin{array}{c} R_{1}=q_{1}\left(p-c\right)=q_{1}\left[A-\left(q_{1}+q_{2}\right)-c\right]=\frac{9{\left(A-c\right)}^{2}}{64},\\ R_{2}=q_{2}\left(p-c\right)=q_{2}\left[A-\left(q_{1}+q_{2}\right)-c\right]=\frac{3{\left(A-c\right)}^{2}}{32}. \end{array}$$

When another company chooses not to cooperate due to its own choice, it still follows the principle of profit maximization:

(8) $$\begin{array}{c} R_{2}=q_{2}\left(p-c\right)=q_{2}\left[A-\left(q_{1}+q_{2}\right)-c\right]. \end{array}$$

The first-order optimal conditions are as follows:

(9) $$\begin{array}{c} \frac{\partial R_{1}}{\partial R_{2}}=A-\left(q_{1}+2q_{2}\right)-c=0. \end{array}$$

Comprehensively, the above two formulas are solved:

(10) $$\begin{array}{c} q_{1}=q_{2}=\frac{A-c}{3},\\ R_{1}=R_{2}=\frac{{\left(A-c\right)}^{2}}{9}. \end{array}$$

Regarding the use of game analysis, if both parties choose to cooperate at this time, they will get the effect of cooperation.

(11) $$\begin{array}{c} K=\frac{{\left(A-c\right)}^{2}}{8}. \end{array}$$

At the same time, for the intention of cooperation, if one party is willing to cooperate and the other party is unwilling to cooperate, for the party who does not want to cooperate:

(12) $$\begin{array}{c} L=\frac{9{\left(A-c\right)}^{2}}{64}. \end{array}$$

Partners get

(13) $$\begin{array}{c} M=\frac{3{\left(A-c\right)}^{2}}{32}. \end{array}$$

If both parties are unwilling to cooperate, then the result is as follows:

(14) $$\begin{array}{c} N=\frac{{\left(A-c\right)}^{2}}{9}. \end{array}$$

It can be concluded that the order of benefits after cooperation between them is as follows: *L* > *K* > *N* > *M*.

If companies conduct multiple comparisons and evaluate the results from multiple aspects, this formula can be derived as follows:

(15) $$\begin{array}{c} K=R+\delta^{2}R+\delta^{3}R+\cdots +\delta^{n}R=\frac{R\left(1-\delta^{n}\right)}{1-\delta }=\frac{{\left(A-c\right)}^{2}}{8\left(1-\delta \right)}. \end{array}$$

The profits gained by the refusal of cooperation between enterprises are as follows:

(16) $$\begin{array}{c} N=\frac{{\left(A-c\right)}^{2}}{9\left(1-\delta \right)}. \end{array}$$

**Figure 1:**
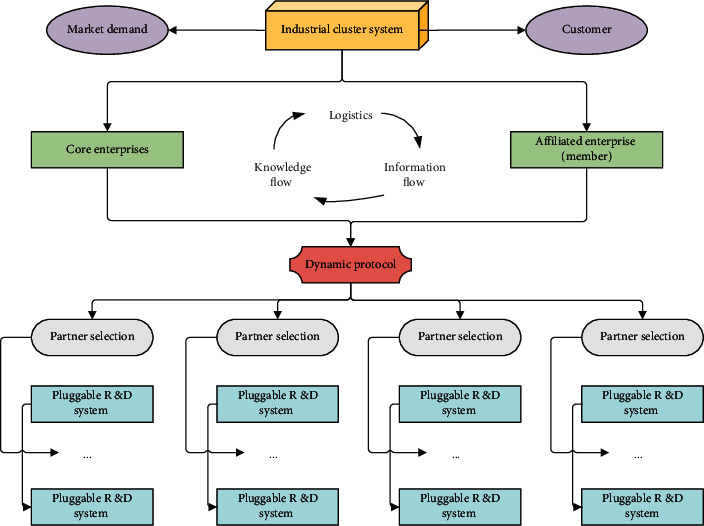
Mechanism of interfirm cooperation in the industrial cluster system.

Therefore, only cooperation is the guarantee of long-term development. For this reason, competition and cooperation within industrial clusters are inevitable. The relationship between the various industries in the process of cooperation is shown in Figure 1.

As shown in Figure 1, the division of labor between different industries in the company is very clear. They let their employees do some major production and development work. The core technology and scientific research work are responsible for the development department. It will be completed by some other companies.

### 3.3. Mathematical Model of Industrial Cluster Learning

#### 3.3.1. Model Establishment

There are two main aspects that have an impact on the industrial production function, namely the total amount of participation and the effective yield. According to the relationship between them, we can build the following functions:

(17) $$\begin{array}{c} Y=E\left(,\right)*F_{p}. \end{array}$$

Different mathematical symbols in the function have different meanings. *E* and *Y*_p_ represent the effective yield and participation amount, respectively. k1 and k2, respectively, represent the private knowledge and learning knowledge in the production process, and *k* represents all the knowledge output, where Y and B represent the functional changes in the learning knowledge and private knowledge in the process, and their scope is *γ*, *θ*∈(0.1). Based on this relationship, we can build the following function:

(18) $$\begin{array}{c} E\left(,\right)=E\left(K_{c},k_{1};\gamma ,\theta \right)=aK_{c}^{y}k_{1}^{\theta }. \end{array}$$

The sample size of the research object is set as *x*, and the total amount of learning knowledge, learning rate, and total enterprise knowledge from these samples are extracted as follows:

(19) $$\begin{array}{c} K_{c}=\int_{0}^{k}k_{2}dt=xk_{2},\\ \pi =k_{2}/k,\\ k=k_{1}+k_{2}. \end{array}$$

FR and *σ* are used to represent the mathematical characteristics of enterprise knowledge under different elements:

(20) $$\begin{array}{c} K_{c}=xk_{2}=x\pi k=K_{c}^{\sigma }F^{R},\\ K_{c}={\left(x\pi F^{R}\right)}^{1}/\left(1-\sigma \right),\\ K_{c}^{y}={\left(x\pi F_{R}\right)}^{y/1-\sigma }. \end{array}$$

Because

(21) $$\begin{array}{c} k_{1}=k-k_{2},\\ k_{1}^{\theta }={\left(k-k_{2}\right)}^{\theta }={\left(1-\pi k\right)}^{\theta }k^{\theta },\\ k^{\theta }={\left(K_{c}^{\sigma }F_{R}\right)}^{\sigma }={\left(x\pi F_{R}\right)}^{\partial \sigma /1-\sigma },\\ k_{1}^{\theta }={\left(1-\pi \right)}^{\theta }k^{\theta }={\left(1-\pi \right)}^{\theta }{\left(x\pi F_{R}\right)}^{\partial \sigma /1-\sigma }. \end{array}$$

Combining equations (19)–(21), these three formulas can be solved:

(22) $$\begin{array}{c} E\left(,\right)=aK_{c}^{y}k_{1}^{\theta }=a{\left(1-\pi \right)}^{\theta }{\left(x\pi F_{R}\right)}^{\lambda },\\ \lambda =\frac{\lambda +\theta \sigma }{1-\sigma }. \end{array}$$

#### 3.3.2. Model Analysis

Because the sample size is fixed, we can establish the following relationships according to the different uses of the input elements:

(23) $$\begin{array}{c} F=F_{P}+F_{R},\\ y=a{\left(1-\pi \right)}^{\theta }{\left(x\pi F_{R}\right)}^{\lambda }F_{R}. \end{array}$$

After establishing the Lagrangian function and solving,

(24) $$\begin{array}{c} L=y-\lambda \left[\left(F_{P}+F_{R}\right)-F\right],\\ \lambda F_{P}=F_{R},F_{R}=F/\left(1+\lambda \right),\\ F_{P}=\lambda F/\left(1+\lambda \right). \end{array}$$

After substituting formula (23),

(25) $$\begin{array}{c} y=A{\left(1-\pi \right)}^{\theta }x^{\lambda }\pi^{\lambda }F_{R}^{1+\lambda },\\ A=a\lambda^{\lambda }{\left(1+\lambda \right)}^{-\left(1+\lambda \right)},\\ \text{if }F=F_{P}+F_{R}=1,\\ y=f\left(\pi ,x\right)=A\phi \left(\pi \right)\varphi \left(x\right),\\ \phi \left(\pi \right)={\left(1-\pi \right)}^{\theta }\pi^{\lambda },\varphi \left(x\right)=x^{\lambda }. \end{array}$$

By analyzing the formula, we can draw the conclusion that there is a positive mathematical relationship between the output of the cooperative group and the knowledge learning rate and the number of enterprises, and the geographical location of the enterprise is also more positive than the output of the cooperative group. The output of the cooperative group mainly depends on the company's technology and knowledge characteristics.

### 3.4. Analysis of Innovation Motivation Mechanism of Industrial Clusters

#### 3.4.1. Path Analysis of Industrial Cluster Learning

**Figure 2:**
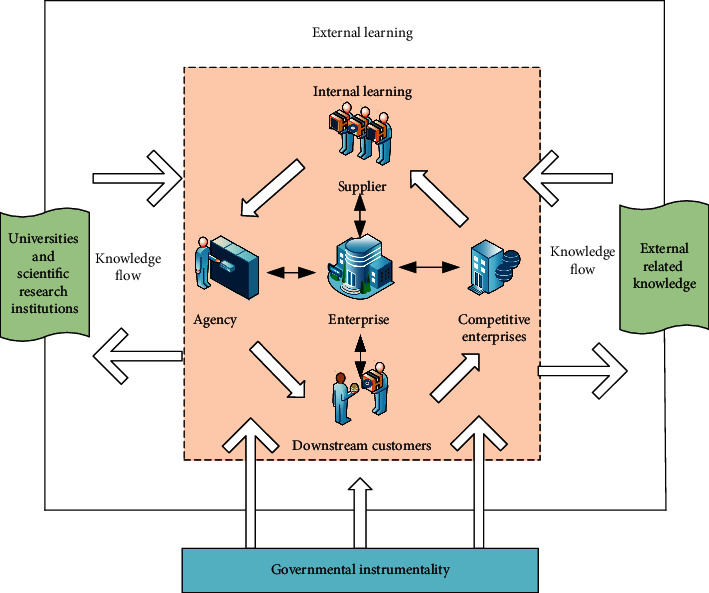
Schematic diagram of the industrial cluster learning system.

The learning system in the industrial cluster is shown in Figure 2.

#### 3.4.2. Industrial Cluster Innovation and Power System

**Figure 3:**
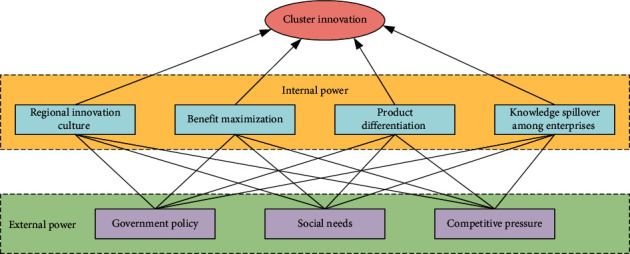
Composition diagram of the main body of industrial cluster innovation.

Innovation can bring development and competition for industrial clusters, and competitors can continuously improve the strength of the enterprise, so innovation is very important between enterprises. Many components can promote the innovation and development of an enterprise. We can roughly divide them into internal and external components. The internal and external components interact and influence each other to form a new driving force to promote the innovation of the cluster, as shown in Figure 3.

## 4. Dairy Industry Cluster Ecosystem Simulation Research Design

### 4.1. Analysis of Dairy Industry Development and Cluster Status

#### 4.1.1. Overview of Price Competition among Dairy Companies

In recent years, the competition among the dairy industries in Western country is mainly reflected in prices. Many companies will reduce prices to increase their competitiveness. Some companies will choose to hold some promotional activities or give away some gifts to improve their competitiveness. In the years of competition, the market value of different dairy industries and the prices of ordinary dairy products will also fluctuate greatly. See Table 1 for specific data.

See Table 2 for analysis of price changes in dairy products in different regions and different brands.

**Figure 4:**
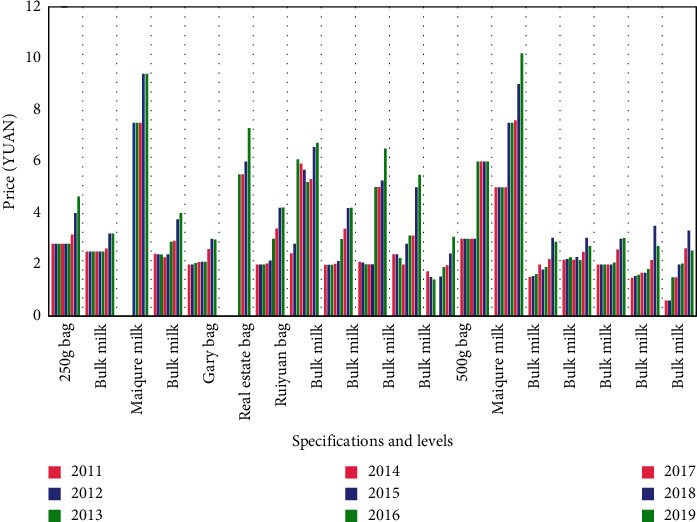
Changes in retail prices of liquid milk in various regions from 2011 to 2019.

The data image corresponding to Table 2 is shown in Figure 4.

#### 4.1.2. Questionnaire Survey-Specific Plan

Many people in China only tasted milk after the founding of New China. Therefore, after the founding of New China, the Chinese country's dairy industry has developed rapidly. Among them, the per capita consumption of dairy products by urban residents in all provinces, cities, and towns across the country in 2013 and 2017 expenses is shown in Table 3.

The data in the table show that the consumption of dairy products in our country is at a relatively low level, not only much lower than some developed countries in Europe and North America, but also much lower than many Asian countries. The per capita consumption of liquid milk in major countries in the world is shown in Table 4.

#### 4.1.3. Group Structure of Dairy Product Consumption

During the research process, the staff sampled the consumption of dairy products by urban and rural residents in a certain province from 2015 to 2017 and studied the differences and mathematical relationships between the consumption of dairy products by residents of different incomes. The specific results are shown in Tables 5 and 6.

### 4.2. The Overall Structure of the Evolution Model of the Industrial Cluster Ecosystem

#### 4.2.1. Ricardo's Rent Subsystem Feedback Loop

**Figure 5:**
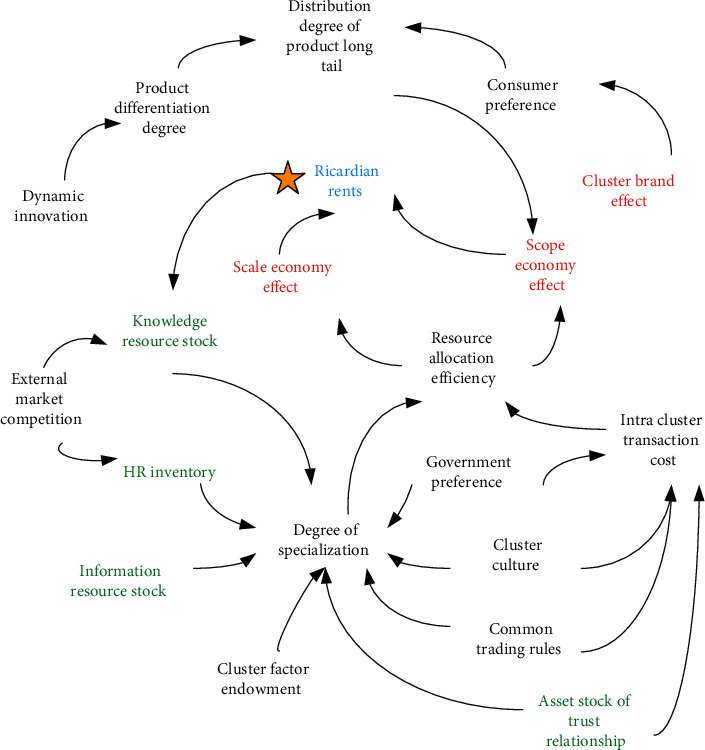
Ricardo's causality diagram of the rent subsystem.

Instead, the enterprise's resource stock, specialization degree of engineering, resource allocation efficiency, etc., are taken. As the research objects, a circular relationship diagram is established, the relationship between them is analyzed, and the analysis results are shown in Figure 5.

The efficiency of resource allocation of enterprises is mainly affected by the specialization of labor and internal exchanges. Staff draw the cause tree to explore the influencing factors of the formation of industrial clusters. Through the observation table, we can intuitively get the relationship between different influencing factors and influencing factors.

### 4.3. The Main Variables and Properties of the Evolution Model of the Industrial Cluster Ecosystem

The rent system of this model and its cluster ecological rent system, the main variables, and types involved are shown in Table 7.

The staff will apply the parameter selection method in the mathematical research to the model to optimize the model, and the specific parameters will be determined after continuous experiments. During the experiment, the staff will make a correction based on the preliminary parameter values obtained. The model is adjusted to a certain extent. If the function of the model does not change significantly before and after the adjustment, then this parameter is the final parameter, as shown in Table 8.

## 5. Simulation Results and Analysis of the Dairy Industry Cluster Innovation Ecosystem

### 5.1. Fitting Test of the Dairy Industry Cluster Ecosystem Evolution Model

The staff will adopt different testing methods to test whether the model is valid and whether the fit of the parameters meets the requirements:

(26) $$\begin{array}{c} R^{2}=1-\frac{\sum_{i=1}^{n}{\left(y_{i}-{\hat{y}}_{l}\right)}^{2}}{\sum_{i=1}^{n}{\left(y_{i}-{\bar{y}}_{l}\right)}^{2}}. \end{array}$$

Different mathematical symbols in the formula represent different meanings.

In this study, industries in a certain area were randomly selected as objects to test the fit. The test process is shown in Table 9.

Table 10 shows that the selected model has practical meaning and is very accurate.

**Figure 6:**
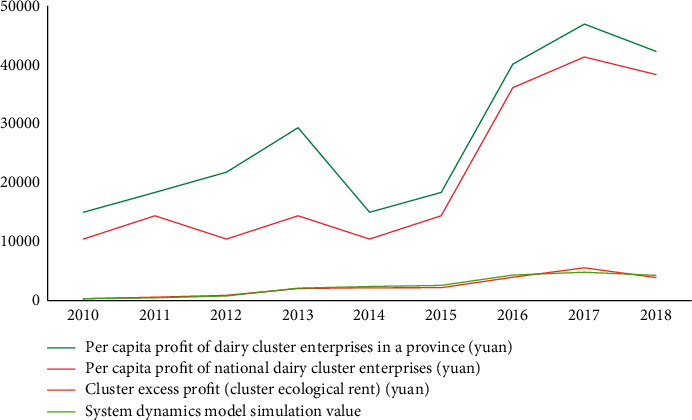
Model mean error analysis bubble chart.

The values in Figure 6 represent the errors generated by the model, and these errors are within the tolerable range, so the model is credible and relatively accurate.

### 5.2. Model Data Extraction and Analysis

Table 11 reflects the changes in the average technical efficiency of my country's dairy industry from 2016 to 2019. Through the table, it is not difficult to see that the per capita efficiency levels of different companies have changed significantly.

Table 12 shows us the distribution of technical efficiency of various dairy companies in my country in 2018.

The staff summarized the approximate level of technical efficiency of my country's dairy companies in 2019, as shown in Table 13.

Based on the above data, the staff has integrated the distribution of the scale of my country's dairy companies in 2019. For detailed data, see Table 14.

The staff collected relevant data on the scale and marketing profit of each dairy industry in 2019 and integrated them into a table (Table 15).

Table 16 shows the calculation status and efficiency of the super-SBM model in each province across the country from 2014 to 2018.

It can be drawn from Table 16 the ranking of super-SBM in various provinces and municipalities across the country from 2014 to 2018.

### 5.3. Simulation Results and Analysis of the Dairy Industry Cluster Ecosystem Evolution Model

The characteristics of the evolution stage of the industrial cluster innovation ecosystem based on simulation are summarized in Table 17.

## 6. Conclusion

With the development of Internet technology, the Internet has played an increasingly important role in industrial clusters, not only affecting the development path of industrial clusters but also changing the development mode of industrial clusters. It can be said that the Internet has a historical role in industrial clusters. In the development process of industrial clusters, there are not only golden opportunities but also severe challenges. We should seize the opportunities without being afraid of the challenges. For industrial clusters, the development of network technology can promote changes in the industrial environment, thereby promoting the development of industrial clusters. In the development process of industrial clusters, the most important thing is practice. Industrial clusters can continuously improve and find and solve problems in practice. Through practice, the development of industrial clusters can be more mature, so that they can adapt to the new environment and welcome new ones. Challenge: the important role of industrial clusters in industrial development has also attracted the attention of many scholars. In recent years, more and more scholars have begun to take industrial clusters as their research direction.

This study mainly analyzes and studies the system and marketing profit level of my country's dairy industry in the form of a combination of research and theory and uses this as a sample to analyze the factors that will have a significant impact on industrial clusters in the process of entrepreneurial cooperation.

The preliminary conclusion drawn from the research is that factors such as government policies, industrial creativity, and industrial scale will all have a greater impact. To promote the long-term development of enterprise clusters, it is necessary to increase innovation and government support.

## Figures and Tables

**Table 1:** Retail price index of Xinjiang dairy products from 1998 to 2009.

Category name	2008	2009	2010	2011	2012	2013	2014	2015	2016	2017	2018	2019	Cumulative increase	Average increase
Dairy products	103.3	101.71	99.6	99.4	99.4	99.9	100.6	98.7	100.8	108.2	122.2	102.0	105.7	102.8
Pasteurized milk	104.9	100.8	100.3	99.7	99 5	100.4	100.3	99.s	100.1	108.4	125.6	101.8	105.7	103.2
Yogurt									103.7	112.4	111.0	101.6	105.7	107.1
Milk powder	98.1	104.4	96.5	97.7	96.2	97.3	99.6	101.4	105.3	102.6	116.3	105.5	105.7	101.6
Other				98.6	100.7	98.3	102.3	93.2	101.3	103.8	106.5	101.4	105.7	100.6

**Table 2:** Retail prices of liquid milk in various regions from 2011 to 2019.

Specification grade	2011	2012	2013	2014	2015	2016	2017	2018	2019
250 g bag	2.80	2.80	2.80	2.80	2.80	2.80	3.16	4.00	4.64
Bulk milk	2.50	2.50	2.50	2.50	2.50	2.50	2.62	3.20	3.20
Maiquer milk					7.50	7.50	7.50	9.40	9.40
Bulk milk	2.42	2.40	2.39	2.28	2.40	2.88	2.92	3.76	4.00
Bulk milk	2.00	2.00	2.06	2.10	2.10	2.11	2.60	3.00	2.97
Gary bag						5.50	5.50	6.00	7.30
Real estate bag	2.00	2.00	2.00	2.04	2.15	3.00	3.40	4.20	4.20
Ruiyuan bag	2.44	2.80	6.08	5.92	5.68	5.20	5.32	6.56	6.72
Bulk milk	2.00	2.00	2.00	2.00	2.00	2.00	2.66	3.50	3.22
Bulk milk	2.10	2.07	2.00	2.00	2.00	5.00	5.00	5.25	6.50
Bulk milk	2.40	2.40	2.25	2.00	2.80	3.12	3.12	5.00	5.48
Bulk milk	1.74	1.51	1.42	1.50	1.53	1.90	1.97	2.43	3.08
500 g bag	3.00	3.00	3.00	3.00	3.00	6.00	6.00	6.00	6.00
Maiquer milk	5.00	5.00	5.00	5.00	7.50	7.50	7.60	9.00	10.15
Bulk milk	1.51	1.55	1.63	2.00	1.80	1.90	2.21	3.04	2.88
Bulk milk	2.17	2.21	2.28	2.18	2.29	2.17	2.49	3.04	2.72
Bulk milk	2.00	2.00	2.00	2.00	2.00	2.08	2.58	3.00	3.03
Bulk milk	1.48	1.55	1.60	1.68	1.68	1.82	2.17	3.50	2.71
Bulk milk	0.60	0.60	1.50	1.50	2.00	2.04	2.61	3.32	2.54

**Table 3:** Per capita consumption expenditure of dairy products by urban residents in various provinces, municipalities, and districts across the country from 2015 to 2019.

Area	2015	2016	2017	2018	2019
National average	124.70	132.37	138.62	15023	160.72
Beijing	255.99	255.79	270.43	279.86	279.45
Tianjin	139.08	135.49	149.17	153.15	182.18
Hebei	117.51	128.16	134.25	140.61	150.19
Shanxi	128.45	14335	154.26	171.83	183.97
Inner Mongolia	109.24	111.99	120.14	124.96	13332
Liaoning	129.81	138_18	140.14	149.13	153.06
Jilin	86.59	102.47	105.30	106.45	120.13
Heilongjiang	101.37	10630	95.60	10633	112.49
Shanghai	241.82	250.03	246.88	267.34	313_04
Jiangsu	141.95	14422	156.29	173.07	18226
Zhejiang	139.17	143.51	155.28	16025	169.31
Female emblem	117.20	137.02	154.65	175.09	192.84
Fujian	153.00	16133	152.34	159.06	164.79
Jiangxi	94.30	104.51	119.63	129.09	157.46
Shandong	149.65	159.16	159.61	176.72	197.63
Henan	91.96	10623	115.26	12322	145.11
Hubei	90.27	97.69	110.25	124.17	143.62
Hunan	87.65	98.52	119.20	117.88	106.98
Guangdong	124.01	127.39	134.49	157.28	147.10
Guangxi	85.31	100.34	99.58	95.03	118.27
Hainan	68.98	85.10	69.53	83.86	104.07
Chongqing	178.21	183.91	168.05	187.78	181.94
Sichuan	130.10	131.07	138.86	146.25	161.74
Guizhou	82.48	86.59	96.40	107.11	118.30
Yunnan	67.73	75.79	78.23	68.82	56.58
Tibet	333.64	419.02	339.90	269.24	284.90
Shaanxi	103.81	112.54	12431	133.19	151.78
Gansu	115.57	125.97	136.80	137.93	140.87
Qinghai	96.29	100.00	104.44	112.77	138.73
Ningxia	103.70	116.89	124.17	135.05	154.82
Xinjiang	110.10	117.28	113.75	123.65	132.77

**Table 4:** Per capita consumption of liquid milk in major countries in the world.

Country	2014	2015	2016	2017	2018	2019
Denmark	133.10	134.00	135.70	136.70	137.20	135.70
France	92.50	92.60	92.50	92.90	91.70	92.20
Germany	89.90	90.10	91.00	93.90	91.90	92.70
Ireland	14420	157.00	157.00	155.70	148.60	
EU 25 countries		90.70	91.70	92.80	92.50	92.80
Netherlands	125.80	120.20	120.50	127.70	127.10	127.10
United Kingdom	114.80	111.80	111.40	111.60	108.90	111.20
Norway	123.40	121.90	121.30	115.20	115.60	114.80
Switzerland	92.50	101.20	106.30	108.50	110.40	111.90
Canada	95.80	94.90	91.80	95.00	95.60	94.50
Mexico	38.10	36.30	39.10	3 9.00	39.80	38.30
United States	87.60	86.30	85.50	84.90	84.50	83.70
Argentina	69.80	70.90	62.20	62.10	65.80	
Brazil	74.60	74.50	71.20	71.00	72.00	73.20
Australia	102.30	103.10	104.00	105.50	106.20	106.30
New Zealand	99.00	98.70	97.00	90.00	90.00	90.00
Japan	3 7.00	35.60	39.20	38.50		
China	1.00	2.20	3.20	5.10	7.20	8.80

**Table 5:** 2017–2019 per capita annual dairy consumption expenditure of urban households in a certain province with different income levels.

Income grouping	2017	2018	2019	Cumulative growth %
Total average (yuan)	113.75	123.65	132.77	8.0
Lowest income households	39.70	54.40	59.21	22.9
Among them, difficult households	23.40	38.75	46.07	42.2
Low-income households	71.58	79.46	101.41	19.3
Middle-lower	96.53	111.50	132.20	17.0
Middle-income households	115.13	120.81	136.41	8.9
Upper-middle class	139.23	144.66	155.05	5.5
High-income households	158.82	182.38	162.30	1.9
Highest income households	207.32	197.61	211.58	12

**Table 6:** 2017-2019 per capita annual dairy consumption expenditure of rural households in a certain province with different income levels.

Income grouping	2017	2018	2019	Cumulative growth (%)
National average (yuan)	9.11	11.35	12.34	25.1
0–400	2.38	7.75	12.40	269.9
400–600	1.10	15.66	10.00	1250.8
600–800	4.00	3.57	4.40	4.6
800–1000	3.55	4.3 9	7.12	75.2
1000–1200	4.80	5.14	3.54	-29.9
1200–1500	7.76	7.41	6.12	-28.1
1500–1700	3.31	7.85	6.28	107.3
1700–2000	6.64	7.93	7.3 6	4.9
2000–2500	9.50	9.44	12.76	25.8
2500–3000	9.04	14.46	8.86	14.3
3000–3500	10.92	17.38	12.22	22.2
3500–4000	18.49	12.84	12.56	-38.9
4000–4500	16.57	17.51	15.72	-11.4
4500–5000	15.08	14.27	13.98	-14.3
>5000	20.79	21.3 7	27.02	20.8

**Table 7:** The main variables of Ricardo's rent system and their properties.

	Variable name	Nature	Initial value
Ricardo rent system	Ricardo rent	Li (t)	0
	Incremental heterogeneous resources	Rl (t)	0.0126
	External market competition	A (t)	0.5
	Stock of knowledge resources	A2 (t)	100
	Human resources stock	a1	100
	Information resource stock	a2	100
	Cluster factor endowment	a3	0.1
	Common trading rules	a4	1

**Table 8:** Main variables and properties of the cluster ecological rent system.

	Variable name	Nature	Initial value
Cluster ecological rent system	Brand devaluation	R4 (t)	0.437
	Leading corporate brand investment	a24	3
	Proprietary resource share	a25	0.137
	Product novelty	A12 (t)	0.245
	Brand effect of leading cluster enterprises	A13 (t)	0.314
	Cluster brand effect	a26	0.56
	Cluster ecological rent	L5 (t)	0
	Competitive advantage	R5 (t)	0.2
	R&D subsidy policy	C1	
	Tax incentives	C2	
	Personnel policy	C3	
	Platform construction policy	C4	
	Brand building policy	C5	

**Table 9:** 2010-2019 China's dairy industry enterprise data.

Year	Total profit of private dairy industry enterprises (10,000 yuan)	Annual average number of employees in private dairy industry enterprises (10,000 people)	Per capita profit of private dairy industry enterprises (yuan)
2010	638200	61.04	10455.44
2011	970900	67.43	14398.64
2012	1399600	80.22	17447.02
2013	1575700	79.81	19743.14
2014	2454200	90.42	27142.23
2015	2691400	84.22	31956.78
2016	3686900	102.065	36123.06
2017	4032000	97.57	41324.18
2018	4362000	113.71	38360.74
2019	4332300	110.92	39057.88

**Table 10:** Validity test of industrial cluster ecosystem system dynamic model.

Year	Per capita profit of dairy cluster enterprises in a province (yuan)	Per capita profit of national dairy cluster enterprises (yuan)	Cluster excess profit (cluster ecological rent) (yuan)	System dynamic model simulation value	Error rate (%)
2010	14998.20	10455.44	333.01	364.44	9.44
2011	18359.25	14398.64	599.56	496.57	−17.18
2012	21803.10	10455.44	912.23	814.14	−10.75
2013	29336.00	14398.64	2059.96	2147.01	4.23
2014	14998.20	10455.44	2193.77	2419.81	10.30
2015	18359.25	14398.64	2204.41	2609.11	18.36
2016	40108.78	36123.06	3985.72	4354.62	9.26
2017	46906.44	41324.18	5582.26	4834.17	−13.40
2018	42296.40	38360.74	3935.66	4298.41	9.22
2019	43023.15 overall goodness-of-fit *R*^2^	39057.88	3965.27	4414.71 91.94%	11.33

**Table 11:** 2016-2019 average technical efficiency of dairy companies.

Index	2015	2016	2017	2018	2019	Mean
Technical efficiency	0.676 6	0.6814	0.7158	0.775 7	0.848 8	0.758 0
Pure technical efficiency	0.764 2	0.784 1	0.8461	0.869 3	0.900 2	0.848 9
Scale efficiency	0.885 4	0.869 0	0.8460	0.892 3	0.9429	0.891 6

**Table 12:** Distribution of technical efficiency of dairy companies in 2018.

Efficiency value distribution	Number of enterprises	Percentage
1	5	13.16
0.9 ≤ *θ* < 1	6	15.79
0.8 ≤ *θ* < 0.9	13	34.21
0.7 ≤ *θ* < 0.8	12	31.58
0.6 ≤ *θ* < 0.7	2	5.26
Total	38	100.00

**Table 13:** Distribution of pure technical efficiency of dairy companies in 2019.

Efficiency value distribution	Number of enterprises	Percentage
1	12	31.58
0.9 ≤ *σ* < 1	7	18.42
0.8 ≤ *σ* < 0.9	12	31.58
0.7 ≤ *σ* < 0.8	7	18.42
Total	38	100.00

**Table 14:** Distribution of scale and efficiency of dairy companies in 2018.

Efficiency value distribution	Number of enterprises	Percentage
1	6	15.79
0.9 ≤ *ω* < 1	23	60.52
0.8 ≤ *ω* < 0.9	6	15.79
0.7 ≤ *ω* < 0.8	3	7.90
Total	38	100.00

**Table 15:** Distribution of changes in returns to scale of dairy companies in 2019.

Scale efficiency	Number of enterprises	Percentage
CRS	11	28.95
IRS	26	68.42
DRS	1	2.63
Total	38	100.00

**Table 16:** Technical efficiency values and rankings calculated by the super-SBM model in various provinces and municipalities across the country from 2015 to 2019.

Area	2015	2016	2017	2018	2019	2015–2019	
	Super-SBM	Super-SBM	Super-SBM	Super-SBM	Super-SBM	Average	Sort
Beijing	0.616 1	0.625 4	0.655 3	0.681 9	0.645 3	0.644 8	15
Tianjin	0.591 1	0.6823	0.596 6	0.633 3	0.6178	0.624 2	19
Hebei	1.1878	1.174 8	1.177 0	0.762 7	1.006 5	1.061 8	2
Shanxi	0.810 0	0.7747	0.601 1	0.698 3	0.575 0	0.6918	12
Inner Mongolia	1.049 3	1.026 7	1.001 4	1.024 4	0.760 0	0.972 3	4
Liaoning	0.546 8	0.552 7	0.653 0	0.737 1	0.930 9	0.684 1	13
Jilin	0.627 7	0.482 9	0.434 5	0.482 7	0.4212	0.489 8	30
Heilongjiang	0.8245	0.809 3	0.771 6	0.837 1	0.861 8	0.820 9	8
Shanghai	1.050 0	1.2228	1.1639	1.159 2	1.372 6	1.193 7	1
Jiangsu	0.698 0	0.658 1	0.599 6	0.618 9	0.610 1	0.637 0	17
Zhejiang	0.6243	0.6286	0.629 4	0.690 6	0.6271	0.640 0	16
Anhui	0.558 6	0.499 0	0.501 2	0.832 6	0.7747	0.633 2	18
Fujian	0.6535	0.552 3	0.521 5	0.640 6	0.602 0	0.594 0	23
Jiangxi	0.6148	0.5976	1.005 6	1.079 8	1.088 4	0.877 3	7
Shandong	0.832 1	0.840 9	0.856 5	1.084 9	1.008 4	0.924 6	5
Henan	0.497 7	0.658 4	0.741 5	0.878 6	0.825 1	0.720 3	10
Hubei	0.583 3	0.590 5	0.5264	0.625 8	0.5514	0.575 5	24
Hunan	1.000 7	0.9110	0.750 5	1.001 1	0.781 7	0.889 0	6
Guangdong	1.051 3	1.0221	1.015 2	1.023 8	1.033 6	1.029 2	3
Guangxi	0.570 2	0.561 2	0.5348	0.597 9	1.003 0	0.653 4	14
Hainan	0.6039	0.553 7	0.547 6	0.597 3	0.5277	0.566 1	26
Chongqing	0.641 8	0.556 0	0.537 4	0.608 2	0.644 8	0.597 6	22
Sichuan	0.611 7	0.626 0	0.640 4	0.487 0	0.648 5	0.6027	21
Guizhou	0.466 7	0.490 8	0.4128	0.419 1	0.412 3	0.440 3	31
Yunnan	0.543 7	0.515 1	0.511 2	0.638 6	0.6320	0.568 1	25
Tibet	0.313 1	1.060 4	1.110 4	0.513 0	0.485 6	0.6965	11
Shaanxi	0.640 0	0.7574	0.704 1	0.843 0	0.8173	0.752 4	9
Gansu	0.520 9	0.5484	0.498 9	0.586 2	0.463 7	0.523 6	28
Qinghai	0.520 8	0.438 9	0.466 8	0.726 4	0.557 3	0.542 0	27
Ningxia	0.6274	0.607 0	0.628 0	0.691 8	0.486 3	0.608 1	20
Xinjiang	0.533 6	0.4924	0.496 6	0.592 5	0.495 4	0.522 1	29
Average	0.6778	0.694 1	0.686 8	0.735 3	0.718 3	0.7025	

**Table 17:** Evolution stage characteristics of the industrial cluster ecosystem based on simulation.

Cluster development history	Segmentation basis	Characteristics of each course
The initial phase	None of the rents appeared	With Ricardo's rent as the mainstay, the advantages of clusters begin to manifest
Progression stage	A small amount of rent	The rents are evenly matched
Perfect stage	The relationship between rents begins to change	Ricardo's rent status has been replaced by the other three rents; the overall level of the cluster is the highest
Metamorphosis stage	Changes in different rent levels	Cluster level decline

## Data Availability

The data used to support the findings of this study are available from the corresponding author upon request.
